# *In Vitro* Antibacterial Activity of Imipenem/Relebactam against Clinical Isolates in Japan

**DOI:** 10.1128/spectrum.02235-21

**Published:** 2022-04-13

**Authors:** Dai Kurihara, Satoru Matsumoto, Naoko Kishi, Yoshikazu Ishii, Masahiko Mori

**Affiliations:** a Japan Development, MSD K.K., Tokyo, Japan; b Microbiological Testing Group, Infectious Diseases Testing Department, Clinical Laboratory Center, Medical Solution Segment, LSI Medience Corporation, Tokyo, Japan; c Department of Microbiology and Infectious Diseases, Toho University School of Medicine, Tokyo, Japan; Emory University School of Medicine

**Keywords:** carbapenem-resistant, imipenem, relebactam, susceptibility

## Abstract

Relebactam is a novel β-lactamase inhibitor of Ambler class A and C β-lactamases that has been developed in combination with imipenem/cilastatin for the treatment of carbapenem-resistant bacterial infections. In this study, we evaluated the *in vitro* antibacterial activity of imipenem/relebactam (IMR) against imipenem-nonsusceptible Enterobacterales and Pseudomonas aeruginosa isolates from Japan. Two sets of antibacterial susceptibility tests were conducted according to the susceptibility testing standard of the Clinical and Laboratory Standards Institute. In the first set, antibacterial susceptibility as measured by the MIC_50/90_ (MIC range) of IMR was assessed for the following 61 imipenem-nonsusceptible strains: 2 Enterobacter cloacae complex (not determined [0.25 μg/mL]), 33 Klebsiella aerogenes (0.5/1 μg/mL [0.5 to 1 μg/mL]), 2 Serratia marcescens (not determined [1 to 2 μg/mL]), and 24 P. aeruginosa (2/128 μg/mL [0.25 to >128 μg/mL]). In the second set, antibacterial susceptibility was assessed for the following 8 imipenem-nonsusceptible strains: 4 Escherichia coli, 1 E. cloacae complex and 3 Klebsiella pneumoniae. The MIC ranges of IMR for these strains were 0.25 to 0.5 μg/mL, 0.5 μg/mL, and 0.5 to 16 μg/mL, respectively. The antibacterial activity of IMR was similar to or lower than that of amikacin and comparable to or greater than those of other reference drugs. In conclusion, IMR has shown antibacterial activity against clinical isolates from Japan and, therefore, is expected to become a new therapeutic option for carbapenem-resistant infections in Japan.

**IMPORTANCE** Carbapenem-resistant Enterobacterales and carbapenem-resistant Pseudomonas aeruginosa strains pose a global threat. Antibacterial activity of imipenem/relebactam (IMR) against clinical isolates of these bacteria from several global regions has been shown; however, as yet there are no reports on Japanese isolates. In this study, we evaluated the *in vitro* antibacterial activity of IMR against imipenem-nonsusceptible Enterobacterales and Pseudomonas aeruginosa isolates from Japan. The antibacterial activity of IMR against imipenem-nonsusceptible Enterobacterales was generally comparable to that of amikacin (AMK) and comparable to or higher than those of other reference drugs tested. The antibacterial activity of IMR against imipenem-nonsusceptible P. aeruginosa isolates was lower than that of AMK but comparable to or higher than those of other drugs. These results support the use of IMR as a new treatment option for infections due to Enterobacterales and P. aeruginosa strains that are resistant to existing β-lactams and other antibacterial agents.

## INTRODUCTION

Relebactam (REL) is a novel, non–β-lactam bicyclic diazabicyclooctane β-lactamase inhibitor that blocks Ambler class A and C β-lactamases like Klebsiella pneumoniae carbapenemase (KPC) and AmpC. The combination drug of REL and imipenem/cilastatin (IMI) has been approved as IMI/REL in the United States, Europe, and Japan. IMI is a combination product of imipenem, a carbapenem antibacterial agent, and cilastatin, a dehydropeptidase-I inhibitor. Imipenem has strong antibacterial activity against Gram-positive and Gram-negative aerobes and anaerobes (1). Since imipenem is inactivated by dehydropeptidase-I in the kidneys, cilastatin is combined with imipenem (1). Cilastatin has no independent antibacterial activity (2). Because of this characteristic, susceptibility tests for IMI/REL are generally conducted using imipenem/relebactam (IMR) without the addition of cilastatin (3).

Gram-negative bacteria cause infections throughout the body, including the bloodstream, respiratory organs, intra-abdominal organs, etc. The emergence of drug-resistant bacteria has become a problem, and in particular, carbapenem-resistant Enterobacterales and carbapenem-resistant Pseudomonas aeruginosa pose a global threat (4). The mechanisms of carbapenem resistance include upregulation of multidrug efflux systems to promote antibacterial expulsion (though imipenem is not subject to efflux [5]), increased production of AmpC or extended-spectrum β-lactamase (ESBL) with concomitant loss of porins that then limits entry of carbapenem into cells (e.g., OprD in the case of imipenem), and production of carbapenemases (6, 7). REL has been shown to restore the antibacterial activity of imipenem against these resistant strains by inhibiting Ambler class A and C β-lactamases, including ESBLs, KPCs, and AmpCs (8). In a global phase 3 study in patients with imipenem-resistant bacterial infections, the efficacy rate for the overall response to IMI/REL was comparable to that of IMI + colistimethate sodium, a comparator drug, and the total mortality was low (9). In Japan, IMI/REL is indicated for treatment of various infections caused by E. coli, Citrobacter spp., Enterobacter spp., Klebsiella spp., Serratia spp., P. aeruginosa, and Acinetobacter spp. that are resistant to carbapenems and susceptible to IMR.

Antibacterial activity of IMR against clinical isolates collected from several global regions has been shown (10, 11); however, as yet there are no reports on Japanese isolates. In this study, the *in vitro* antibacterial activity of IMR against clinical isolates collected from Japan was investigated in 2 analyses. The first was a set of susceptibility tests using clinical isolates collected prospectively from medical institutions across Japan in 2016. A part of the results was previously reported by Hidaka et al. (12), but the current study reports the antibacterial activity of IMR and antibacterial susceptibility of imipenem-nonsusceptible strains. The second analysis involves antibacterial susceptibility testing of imipenem-nonsusceptible bacterial stocks. In both tests, the antibacterial activity of IMR was compared with that of control drugs to identify the *in vitro* antibacterial profiles against clinical isolates from Japan.

## RESULTS

In this prospective antibacterial susceptibility study, 850 isolates of species listed in the indication for IMI/REL (approved in 2021 in Japan) were collected, of which 2 Enterobacter cloacae complex isolates, 33 Klebsiella aerogenes isolates, 2 Serratia marcescens isolates, and 24 P. aeruginosa isolates were imipenem-nonsusceptible. All of the E. cloacae complex isolates and K. aerogenes isolates were imipenem-intermediate, and there were no imipenem-resistant isolates. Minimum inhibitory concentrations (MIC) were interpreted as susceptible, intermediate, and resistant using Clinical and Laboratory Standards Institute (CLSI) breakpoints (3). Of the 2 S. marcescens isolates, 1 was imipenem intermediate and the other was imipenem resistant. Three of the 24 P. aeruginosa isolates were imipenem intermediate, and 21 of 24 were imipenem resistant. The antibacterial activities of IMR, imipenem, and reference drugs (meropenem [MEM], tazobactam/piperacillin [TZP], ceftazidime [CAZ], cefepime [FEP], levofloxacin [LVX], amikacin [AMK], colistin [CST], and tigecycline [TGC]) against these strains are shown in Table 1.

**TABLE 1:** Antibacterial activity of IMR and other agents against imipenem-nonsusceptible isolates collected in the prospective study

Species	No. of isolates	Antibacterial agent^*a*^	MIC (μg/mL)			No. (%) with MIC interpretation^*b*^:		
			Range	MIC_50_	MIC_90_	Susceptible	Intermediate	Resistant
Enterobacter cloacae complex	2	IMR	0.25	—^*c*^	—	2 (100.0)	0	0
		Imipenem	2	—	—	0	2 (100.0)	0
		MEM	≤0.06	—	—	2 (100.0)	0	0
		TZP	0.5 to 4	—	—	2 (100.0)	0	0
		CAZ	0.25 to 0.5	—	—	2 (100.0)	0	0
		FEP	≤0.06 to 0.12	—	—	2 (100.0)	0^*d*^	0
		LVX	≤0.06	—	—	2 (100.0)	0	0
		AMK	1 to 2	—	—	2 (100.0)	0	0
		CST	>32	—	—	—	0	2 (100.0)
		TGC	0.5 to 2	—	—	1 (50.0)	—	1 (50.0)

Klebsiella aerogenes	33	IMR	0.5 to 1	0.5	1	33 (100.0)	0	0
		Imipenem	2	2	2	0	33 (100.0)	0
		MEM	≤0.06 to 0.12	≤0.06	≤0.06	33 (100.0)	0	0
		TZP	2 to 64	2	16	30 (90.9)	3 (9.1)	0
		CAZ	0.12 to 64	0.5	2	30 (90.9)	0	3 (9.1)
		FEP	≤0.06 to 0.25	≤0.06	0.12	33 (100.0)	0^*d*^	0
		LVX	≤0.06 to 1	≤0.06	0.12	32 (97.0)	1 (3.0)	0
		AMK	1 to 4	2	2	33 (100.0)	0	0
		CST	0.5 to 2	1	1	—	33 (100.0)	0
		TGC	0.5 to 8	0.5	1	21 (63.6)	—	12 (36.4)

Serratia marcescens	2	IMR	1 to 2	—	—	1 (50.0)	1 (50.0)	0
		Imipenem	2 to 16	—	—	0	1 (50.0)	1 (50.0)
		MEM	≤0.06 to 2	—	—	1 (50.0)	1 (50.0)	0
		TZP	8 to 16	—	—	2 (100.0)	0	0
		CAZ	0.5 to 8	—	—	1 (50.0)	1 (50.0)	0
		FEP	0.12 to 8	—	—	1 (50.0)	1 (50.0)^*d*^	0
		LVX	≤0.06 to 16	—	—	1 (50.0)	0	1 (50.0)
		AMK	2 to 4	—	—	2 (100.0)	0	0
		TGC	1 to 4	—	—	0	—	2 (100.0)

Pseudomonas aeruginosa	24	IMR	0.25 to >128	2	128	13 (54.2)	3 (12.5)	8 (33.3)
		Imipenem	4 to >128	16	128	0	3 (12.5)	21 (87.5)
		MEM	1 to >128	32	>128	4 (16.7)	3 (12.5)	17 (70.8)
		TZP	0.5 to >128	32	128	10 (41.7)	9 (37.5)	5 (20.8)
		CAZ	1 to >128	8	>128	12 (50.0)	1 (4.2)	11 (45.8)
		FEP	0.5 to >128	8	>128	13 (54.2)	3 (12.5)	8 (33.3)
		LVX	0.5 to >128	16	>128	3 (12.5)	2 (8.3)	19 (79.2)
		AMK	1 to >128	4	128	19 (79.2)	1 (4.2)	4 (16.7)
		CST	1 to 2	2	2	—	24 (100.0)	0
		TGC	4 to 64	16	64	—	—	—

a AMK, amikacin; FEP, cefepime; CAZ, ceftazidime; CST, colistin; IMR, imipenem/relebactam; LVX, levofloxacin; MEM, meropenem; TZP, tazobactam/piperacillin; TGC, tigecycline.

b For TGC against Enterobacterales, European Committee on Antimicrobial Susceptibility Testing (EUCAST) 2021 breakpoints were applied because Clinical and Laboratory Standards Institute (CLSI) breakpoints were not defined.

c —, Not applicable.

d Susceptible, dose-dependent.

The isolates of E. cloacae complex and K. aerogenes were all susceptible to IMR. The MIC of IMR against E. cloacae complex isolates was 0.25 μg/mL, 8-fold lower than that of imipenem. The MIC_50/90_ of IMR against K. aerogenes was 0.5/1 μg/mL, 2- to 4-fold lower than that of imipenem. The antibacterial activities of most reference drugs against these strains were comparable to those of IMR; the MIC_50/90_ values were low and the percentages of susceptibility were high. As an exception, the MIC of CST against imipenem-nonsusceptible E. cloacae complex isolates was high (>32 μg/mL) and 2 of 2 isolates were resistant to CST. In addition, 36.4% of imipenem-nonsusceptible K. aerogenes isolates were resistant to TGC.

The MICs of imipenem against 2 imipenem-nonsusceptible strains of S. marcescens were 2 μg/mL and 16 μg/mL, while the MICs of IMR were 1 μg/mL and 2 μg/mL, respectively, 2- to 8-fold lower than those of imipenem alone. One isolate was imipenem-resistant and another was imipenem-intermediate. As for the reference drugs, both isolates were susceptible to TZP and AMK and resistant to TGC. For the other drugs, the antibacterial activities were generally similar to those of IMR.

The MIC_50_ of IMR against imipenem-nonsusceptible P. aeruginosa isolates was 2 μg/mL, 8-fold lower than that of imipenem. The MIC_90_ of IMR was 128 μg/mL, equivalent to that of imipenem. The percentage of susceptibility to IMR was 54.2%, showing a large decrease in the resistance rate and an increase in the susceptibility rate compared with those of imipenem. The MIC_90_ values of the reference drugs were also high, except for CST. The percentage of susceptibility was the highest for AMK at 79.2%, while the percentages of susceptibility to MEM, TZP, and LVX were relatively low, and the percentages of susceptibility to CAZ and FEP were comparable to that of IMR.

Since the imipenem-nonsusceptible strains collected in the prospective study only covered some of the bacterial species listed in the indication for IMI/REL, a second set of antibacterial susceptibility tests was performed using the following existing stocks of imipenem-nonsusceptible clinical isolates: 4 E. coli, 1 E. cloacae complex and 3 K. pneumoniae. The antibacterial activities of IMR, imipenem, and the reference drugs against these strains are shown in Table 2.

**TABLE 2:** Antibacterial activity of IMR and other agents against preserved imipenem-nonsusceptible isolates

Species	No. of isolates	Antibacterial agent^*a*^	MIC range (μg/mL)	No. (%) with MIC interpretation^*b*^:		
				Susceptible	Intermediate	Resistant
Escherichia coli	4	IMR	0.2 to 0.5	4 (100.0)	0	0
		Imipenem	2 to 8	0	2 (50.0)	2 (50.0)
		MEM	4 to 8	0	0	4 (100.0)
		TZP	128 to >128	0	0	4 (100.0)
		CAZ	128 to >128	0	0	4 (100.0)
		FEP	8 to >128	0	1 (25.0)^*c*^	3 (75.0)
		LVX	16 to 32	0	0	4 (100.0)
		AMK	2 to 4	4 (100.0)	0	0
		CST	1	—^*d*^	4 (100.0)	0
		TGC	0.25 to 0.5	4 (100.0)	—	0

Enterobacter cloacae complex	1	IMR	0.5	1 (100.0)	0	0
		Imipenem	8	0	0	1 (100.0)
		MEM	8	0	0	1 (100.0)
		TZP	>128	0	0	1 (100.0)
		CAZ	>128	0	0	1 (100.0)
		FEP	>128	0	0^*c*^	1 (100.0)
		LVX	2	0	0	1 (100.0)
		AMK	1	1 (100.0)	0	0
		CST	1	—	1 (100.0)	0
		TGC	1	0	—	1 (100.0)

Klebsiella pneumoniae	3	IMR	0.5 to 16	1 (33.3)	1 (33.3)	1 (33.3)
		Imipenem	2 to 32	0	1 (33.3)	2 (66.7)
		MEM	16	0	0	3 (100.0)
		TZP	16 to >128	1 (33.3)	0	2 (66.7)
		CAZ	4 to >128	1 (33.3)	0	2 (66.7)
		FEP	1 to >128	1 (33.3)	0^*c*^	2 (66.7)
		LVX	0.12 to >128	1 (33.3)	0	2 (66.7)
		AMK	2 to >128	2 (66.7)	0	1 (33.3)
		CST	0.5 to 1	—	3 (100.0)	0
		TGC	0.25 to 1	2 (66.6)	—	1 (33.3)

a AMK, amikacin; FEP, cefepime; CAZ, ceftazidime; CST, colistin; IMR, imipenem/relebactam; LVX, levofloxacin; MEM, meropenem; TZP, tazobactam/piperacillin; TGC, tigecycline.

b For TGC against Enterobacterales, EUCAST breakpoints in 2021 were applied because CLSI breakpoints were not defined.

c Susceptible, dose-dependent.

d —, Not applicable.

The MICs of IMR against imipenem-nonsusceptible E. coli isolates were 0.25 to 0.5 μg/mL, 4- to 32-fold lower than those of imipenem. All 4 strains were susceptible to IMR, AMK, and TGC, intermediate to CST, and resistant to almost all other reference drugs. In particular, the MICs of TZP and CAZ were as high as 128 to >128 μg/mL.

The MIC of IMR against the imipenem-nonsusceptible E. cloacae complex isolate was 0.5 μg/mL, 16-fold lower than that of imipenem. This strain was susceptible to IMR and AMK and intermediate to CST but resistant to other drugs. In particular, the MICs of TZP, CAZ, and FEP were as high as >128 μg/mL.

The MIC range of IMR against imipenem-nonsusceptible K. pneumoniae isolates was 0.5 to 16 μg/mL, 2- to 8-fold lower than the MIC range of imipenem. The number of isolates susceptible, intermediate, and resistant to IMR was 1 each. For the reference drugs AMK and TGC, 2 isolates and 1 isolate were susceptible and resistant, respectively, and all were intermediate to CST. All isolates were resistant to MEM, and 2 of 3 isolates were resistant to other drugs. One imipenem-nonsusceptible K. pneumoniae strain was resistant to all drugs except CST, and the MICs were very high (multidrug-resistant strain).

Table 3 shows the antibacterial activities of IMR, imipenem, and the reference drugs against imipenem-nonsusceptible strains of species listed in the indication for IMI/REL for which β-lactamases were identified in the 2 studies. The numbers of AmpC-positive imipenem-nonsusceptible strains obtained in the 2 studies were as follows: 1 for E. coli, 3 for K. aerogenes, 1 for K. pneumoniae, and 1 for S. marcescens. In addition, 5 strains of P. aeruginosa were found to be constitutive producers of AmpC. The K. pneumoniae isolate was the multidrug-resistant strain described above. Among these 11 isolates, 1 E. coli isolate, 3 K. aerogenes isolates, and 4 P. aeruginosa isolates were susceptible to IMR. The MICs of IMR were lower than those of imipenem even in isolates that were not susceptible to IMR (data not shown), which is consistent with previous reports (5, 13, 14). Among the reference drugs, AMK showed a higher antibacterial activity than IMR. All but 1 K. pneumoniae isolate were susceptible to AMK. All isolates were intermediate to CST. The other reference drugs had lower antibacterial activities against P. aeruginosa, and only 1 isolate was susceptible to each drug. The antibacterial activities of TZP and CAZ against constitutive AmpC-producing isolates were generally low regardless of species, with 18.2% of AmpC-producing isolates susceptible to TZP (S. marcescens, *N* = 1; P. aeruginosa, *N* = 1) and 9.1% susceptible to CAZ (P. aeruginosa, *N* = 1).

**TABLE 3:** β-Lactamase profiles and antibiograms of imipenem-nonsusceptible isolates^*a*^

β-Lactamase	Species	No. of isolates:		MIC interpretation (susceptible/intermediate/resistant)^*b*^										Data source
		Tested	Positive for *β*-lactamase tested	IMR	Imipenem	MEM	TZP	CAZ	FEP	LVX	AMK	CST	TGC	
AmpC	Escherichia coli	4	1	1/0/0	0/0/1	0/0/1	0/0/1	0/0/1	0/1^*c*^/0	0/0/1	1/0/0	—^*d*^/1/0	1/—/0	Bacterial stocks
	Klebsiella aerogenes	33	3^*e*^	3/0/0	0/3/0	3/0/0	0/3/0	0/0/3	3/0^*c*^/0	2/1/0	3/0/0	—/3/0	2/—/1	Prospective study
	Klebsiella pneumoniae	3	1	0/0/1	0/0/1	0/0/1	0/0/1	0/0/1	0/0^*c*^/1	0/0/1	0/0/1	—/1/0	0/—/1	Bacterial stocks
	Serratia marcescens	2	1^*e*^	0/1/0	0/0/1	0/1/0	1/0/0	0/1/0	0/1^*c*^/0	0/0/1	1/0/0	—	0/—/1	Prospective study
	Pseudomonas aeruginosa	24	5^*e*^	4/1/0	0/0/5	1/0/4	1/1/3	1/0/4	1/2/2	1/0/4	5/0/0	—/5/0	—	Prospective study

ESBL	E. coli	4	3	3/0/0	0/2/1	0/0/3	0/0/3	0/0/3	0/0^*c*^/3	0/0/3	3/0/0	—/3/0	3/—/0	Bacterial stocks
	K. pneumoniae	3	1	1/0/0	0/1/0	0/0/1	0/0/1	0/0/1	0/0^*c*^/1	0/0/1	1/0/0	—/1/0	1/—/0	Bacterial stocks

GES-type carbapenemase	K. pneumoniae	3	1	0/1/0	0/0/1	0/0/1	1/0/0	1/0/0	1/0^*c*^/0	1/0/0	1/0/0	—/1/0	1/—/0	Bacterial stocks

MBL	P. aeruginosa	24	6	0/0/6	0/0/6	0/0/6	2/3/1	0/0/6	0/0/6	0/0/6	1/1/4	—/6/0	—	Prospective study

a AMK, amikacin; FEP, cefepime; CAZ, ceftazidime; CST, colistin; ESBL, extended-spectrum β-lactamase; GES, Guiana extended spectrum; IMR, imipenem/relebactam; LVX, levofloxacin; MEM, meropenem; MBL, metallo-β-lactamase; MIC, MIC; TZP, tazobactam/piperacillin; TGC, tigecycline.

b For TGC against Enterobacterales, EUCAST breakpoints in 2021 were applied because CLSI breakpoints were not defined.

c Susceptible, dose-dependent.

d —, Not applicable.

e Isolate(s) constitutively express AmpC.

ESBL-positive imipenem-nonsusceptible strains comprised 3 E. coli isolates and 1 K. pneumoniae isolate. All 4 of these isolates possessed CTX-M-type ESBLs and were susceptible to IMR. As for the reference drugs, the isolates were all susceptible to AMK and TGC, intermediate to CST, and resistant to the others. One imipenem-nonsusceptible K. pneumoniae isolate was positive for the Guiana extended-spectrum (GES)–type carbapenemase and was intermediate to IMR. This isolate was resistant to MEM, intermediate to CST, and susceptible to other drugs. Six imipenem-nonsusceptible P. aeruginosa isolates possessed metallo-β-lactamases (MBLs). These 6 isolates were resistant to all drugs, including IMR, except for TZP, AMK, and CST. The numbers of isolates susceptible to TZP and AMK were 2 and 1, respectively, and all were intermediate to CST.

The antibacterial activities of IMR and imipenem against isolates of species listed in the Japanese indication for IMI collected in the prospective study are shown in Table 4. Overall, the rate of susceptibility of aerobic Gram-negative bacteria to IMR was high. The MIC_90_ range of IMR in Enterobacterales other than *Morganellaceae* was 0.12 to 1 μg/mL, indicating that almost all strains were susceptible to IMR. Among them, no imipenem-nonsusceptible isolates were collected for Citrobacter spp. and E. coli; however, the MIC_50_ and MIC_90_ values of IMR for these isolates were lower than those of imipenem (13, 15). The MIC_50/90_ values of IMR for isolates of P. aeruginosa and Acinetobacter spp., the other species listed in the indication for IMI/REL, were 0.25/4 μg/mL and 0.12/0.25 μg/mL, respectively, and the percentages of susceptibility to IMR were 89.0% and 100%, respectively. In the *Morganellaceae* (Morganella morganii, Proteus mirabilis, Proteus vulgaris, and Providencia spp.), there were imipenem-nonsusceptible isolates, but enhancement of the antibacterial activity of imipenem by REL was nearly absent except for M. morganii. The Haemophilus influenzae isolates were 100% susceptible to IMR and imipenem.

**TABLE 4:** Antibacterial activity of IMR and other agents against isolates collected in this prospective study

Species^*a*^	No. of isolates	Antibacterial agent^*b*^	MIC (μg/mL)			No. (%) with MIC interpretation		
			Range	MIC_50_	MIC_90_	Susceptible	Intermediate	Resistant
Gram-positive aerobes								
MSSA	50	IMR	≤0.06	≤0.06	≤0.06	—^*c*^	—	—
		Imipenem	≤0.06	≤0.06	≤0.06	—	—	—
MRSA	50	IMR	≤0.06 to 64	≤0.06	32	—	—	—
		Imipenem	≤0.06 to 64	≤0.06	32	—	—	—
MSSE	20	IMR	≤0.06	≤0.06	≤0.06	—	—	—
		Imipenem	≤0.06	≤0.06	≤0.06	—	—	—
MRSE	50	IMR	≤0.06 to 32	0.12	4	—	—	—
		Imipenem	≤0.06 to 32	0.12	4	—	—	—
MSCNS	20	IMR	≤0.06	≤0.06	≤0.06	—	—	—
		Imipenem	≤0.06	≤0.06	≤0.06	—	—	—
MRCNS	50	IMR	≤0.06 to 128	0.12	64	—	—	—
		Imipenem	≤0.06 to 128	0.12	64	—	—	—
Enterococcus faecalis	50	IMR	0.5 to 4	1	1	—	—	—
		Imipenem	0.5 to 4	1	1	—	—	—
Enterococcus faecium	50	IMR	128 to >128	>128	>128	—	—	—
		Imipenem	128 to >128	>128	>128	—	—	—
Enterococcus avium	25	IMR	0.5 to 32	2	8	—	—	—
		Imipenem	0.5 to 32	2	8	—	—	—
PSSP	25	IMR	≤0.06	≤0.06	≤0.06	25 (100.0)^*d*^	0^*d*^	0^*d*^
		Imipenem	≤0.06	≤0.06	≤0.06	25 (100.0)	0	0
PRSP	25	IMR	0.25 to 0.5	0.25	0.5	0^*d*^	25 (100.0)^*d*^	0^*d*^
		Imipenem	0.25 to 0.5	0.25	0.5	0	25 (100.0)	0
Streptococcus pyogenes	50	IMR	≤0.06	≤0.06	≤0.06	—	—	—
		Imipenem	≤0.06	≤0.06	≤0.06	—	—	—
Streptococcus agalactiae	50	IMR	≤0.06	≤0.06	≤0.06	—	—	—
		Imipenem	≤0.06	≤0.06	≤0.06	—	—	—
Streptococcus mitis group	20	IMR	≤0.06	≤0.06	≤0.06	—	—	—
		Imipenem	≤0.06	≤0.06	≤0.06	—	—	—
Streptococcus anginosus	20	IMR	≤0.06	≤0.06	≤0.06	—	—	—
		Imipenem	≤0.06	≤0.06	≤0.06	—	—	—
Streptococcus constellatus	20	IMR	≤0.06	≤0.06	≤0.06	—	—	—
		Imipenem	≤0.06	≤0.06	≤0.06	—	—	—
Streptococcus salivarius	10	IMR	≤0.06	≤0.06	≤0.06	—	—	—
		Imipenem	≤0.06	≤0.06	≤0.06	—	—	—
Staphylococcus intermedius	10	IMR	≤0.06	≤0.06	≤0.06	—	—	—
		Imipenem	≤0.06	≤0.06	≤0.06	—	—	—

Gram-negative aerobes								
* *Citrobacter spp.	100	IMR	0.12 to 1	0.12	0.25	100 (100.0)	0	0
		Imipenem	0.12 to 1	0.25	1	100 (100.0)	0	0
Escherichia coli	100	IMR	≤0.06 to 1	0.12	0.12	100 (100.0)	0	0
		Imipenem	≤0.06 to 1	0.12	0.25	100 (100.0)	0	0
Enterobacter cloacae complex	100	IMR	0.12 to 0.5	0.25	0.25	100 (100.0)	0	0
		Imipenem	0.12 to 2	0.5	1	98 (98.0)	2 (2.0)	0
Klebsiella pneumoniae	100	IMR	≤0.06 to 1	0.12	0.5	100 (100.0)	0	0
		Imipenem	≤0.06 to 1	0.12	0.5	100 (100.0)	0	0
Klebsiella oxytoca	100	IMR	0.12 to 0.25	0.12	0.25	100 (100.0)	0	0
		Imipenem	0.12 to 0.25	0.12	0.25	100 (100.0)	0	0
Klebsiella aerogenes	100	IMR	0.12 to 1	0.5	1	100 (100.0)	0	0
		Imipenem	0.25 to 2	1	2	67 (67.0)	33 (33.0)	0
Serratia marcescens	100	IMR	0.12 to 2	0.5	1	99 (99.0)	1 (1.0)	0
		Imipenem	0.25 to 16	0.5	1	98 (98.0)	1 (1.0)	1 (1.0)
Morganella morganii	100	IMR	0.5 to 2	1	2	60 (60.0)^*d*^	40 (40.0)^*d*^	0^*d*^
		Imipenem	0.5 to 4	2	2	35 (35.0)	60 (60.0)	5 (5.0)
Proteus mirabilis	100	IMR	≤0.06 to 4	0.5	2	85 (85.0)^*d*^	13 (13.0)^*d*^	2 (2.0)^*d*^
		Imipenem	≤0.06 to 4	0.5	2	85 (85.0)	12 (12.0)	3 (3.0)
Proteus vulgaris	100	IMR	≤0.06 to 2	1	2	71 (71.0)^*d*^	29 (29.0)^*d*^	0^*d*^
		Imipenem	0.12 to 4	1	2	69 (69.0)	30 (30.0)	1 (1.0)
Providencia spp.	100	IMR	0.5 to 4	1	2	81 (81.0)^*d*^	17 (17.0)^*d*^	2 (2.0)^*d*^
		Imipenem	0.5 to 4	1	2	81 (81.0)	17 (17.0)	2 (2.0)
Pseudomonas aeruginosa	100	IMR	0.12 to >128	0.25	4	89 (89.0)	3 (3.0)	8 (8.0)
		Imipenem	0.5 to >128	2	32	76 (76.0)	3 (3.0)	21 (21.0)
Acinetobacter spp.	50	IMR	≤0.06 to 0.5	0.12	0.25	50 (100.0)^*d*^	0^*d*^	0^*d*^
		Imipenem	≤0.06 to 1	0.12	0.25	50 (100.0)	0	0
Burkholderia cepacia complex	25	IMR	≤0.06 to 4	0.25	1	—	—	—
		Imipenem	1 to 16	4	8	—	—	—
BLNAR	25	IMR	0.25 to 4	1	2	25 (100.0)^*d*^	—	—
		Imipenem	0.25 to 4	1	2	25 (100.0)	—	—
Haemophilus influenzae (other than BLNAR)	25	IMR	≤0.06 to 2	0.5	1	25 (100.0)^*d*^	—	—
		Imipenem	≤0.06 to 2	0.5	1	25 (100.0)	—	—

Anaerobes								
* *Peptostreptococcus spp.	25	IMR	≤0.06	≤0.06	≤0.06	25 (100.0)	0	0
		Imipenem	≤0.06	≤0.06	≤0.06	25 (100.0)	0	0
* *Bacteroides spp.	25	IMR	≤0.06 to 8	0.25	1	24 (96.0)	1 (4.0)	0
		Imipenem	≤0.06 to 16	0.25	2	24 (96.0)	0	1 (4.0)
* *Prevotella spp.	25	IMR	≤0.06 to 0.12	≤0.06	≤0.06	25 (100.0)	0	0
		Imipenem	≤0.06 to 0.12	≤0.06	≤0.06	25 (100.0)	0	0

a BLNAR, β-lactamase–negative ampicillin-resistant H. influenzae; MSCNS, methicillin-susceptible coagulase-negative Staphylococcus (other than Staphylococcus epidermidis); MSSA, methicillin-susceptible Staphylococcus aureus; MSSE, methicillin-susceptible S. epidermidis; MRCNS, methicillin-resistant coagulase-negative Staphylococcus (other than S. epidermidis); MRSE, methicillin-resistant S. epidermidis; MRSA, methicillin-resistant S. aureus; PSSP, penicillin-susceptible Streptococcus pneumoniae; PRSP, penicillin-resistant S. pneumoniae.

b IMR, imipenem/relebactam.

c —, Not applicable.

d Calculated according to imipenem breakpoints because breakpoints for IMR were not defined.

The antibacterial activities of IMR against aerobic Gram-positive bacteria were similar to those of imipenem. While the MIC_90_ values of IMR and imipenem were high for methicillin-resistant Staphylococcus aureus (MRSA), methicillin-resistant Staphylococcus epidermidis (MRSE), methicillin-resistant coagulase-negative Staphylococcus (MRCNS; other than S. epidermidis), Enterococcus faecium, and Enterococcus avium isolates, those for the other species were low (≤0.06 to 1 μg/mL).

Anaerobic bacteria were highly susceptible to IMR. The MIC_50/90_ values for Peptostreptococcus spp. and Prevotella spp. were ≤0.06/≤0.06 μg/mL, and the percentage of susceptibility was 100%. The MIC_50/90_ for Bacteroides spp. was 0.25/1 μg/mL, and the percentage of susceptibility was 96.0%. Enhancement of imipenem antibacterial activity by REL was observed in Bacteroides spp.

## DISCUSSION

In this study, the antibacterial activities of IMR and reference drugs against clinical isolates from Japan were evaluated in 2 analyses. Among the species listed in the indication for IMI/REL, imipenem-nonsusceptible isolates of E. coli, E. cloacae complex, K. pneumoniae, K. aerogenes, S. marcescens, and P. aeruginosa were collected. Overall, the susceptibility of these isolates to IMR was high. All isolates of E. coli, E. cloacae complex, and K. aerogenes were susceptible to IMR. Based on the CLSI breakpoint for IMR (MIC of ≤1 μg/mL) (3), there were IMR-nonsusceptible isolates of K. pneumoniae, S. marcescens, and P. aeruginosa. Of these isolates, the MIC of 1 K. pneumoniae isolate and 1 S. marcescens isolate was 2 μg/mL, which is considered intermediate susceptibility according to the CLSI breakpoint for IMR (MIC of 2 μg/mL). However, it has been reported that at the recommended IMI/REL dosing regimens, >90% of patients were predicted to achieve joint pharmacokinetic/pharmacodynamic targets at an MIC breakpoint of ≤2 μg/mL, which aligns with the European Committee on Antimicrobial Susceptibility Testing (EUCAST) breakpoint for IMR (16, 17). Taking this into consideration, IMR may be effective for 2/3 and 2/2 of imipenem-nonsusceptible isolates of K. pneumoniae and S. marcescens collected in this study, respectively, and thus, these isolates would be considered susceptible by EUCAST breakpoints but of intermediate susceptibility by CLSI breakpoints.

Comparison of the antibacterial activities of IMR with those of the reference drugs showed that the antibacterial activities of IMR against imipenem-nonsusceptible Enterobacterales were generally comparable to those of AMK and comparable to or higher than those of other drugs. In particular, the differences between IMR and MEM, TZP, CAZ, and FEP were notable for isolates with low susceptibility to imipenem. The antibacterial activities of IMR against imipenem-nonsusceptible P. aeruginosa isolates were lower than that of AMK and comparable to or higher than those of other drugs. The percentages of susceptibility to CST were intermediate for both imipenem-nonsusceptible Enterobacterales and P. aeruginosa isolates. CLSI does not define a susceptibility breakpoint for CST; however, pharmacokinetic/pharmacodynamic analysis has shown that less than 50% of patients can achieve the blood concentrations of CST required for killing bacteria due to the high risk of nephrotoxicity (18). These results indicate that IMR can be a new treatment option for Enterobacterales and P. aeruginosa isolates that are resistant to existing β-lactams and other antibacterial agents.

The antibacterial activities against imipenem-nonsusceptible isolates possessing β-lactamases were also assessed. The antibacterial activities of IMR were lower than those of AMK and higher than those of the other reference drugs against isolates with AmpC, and were similar to those of AMK and TGC, and higher than those of the other reference drugs against isolates with ESBL. As the production of AmpC or ESBL concomitant with entry porin deficiency is a known mechanism of carbapenem resistance (6, 7), these results suggest the potential utility of IMR against bacteria that are carbapenem resistant due to these mechanisms.

In 2 sets of antibacterial susceptibility tests, 8 P. aeruginosa isolates and 1 K. pneumoniae isolate were resistant to IMR. Six of 8 IMR-resistant P. aeruginosa strains possessed an MBL. Since REL does not inhibit MBL (19), this result was expected. The IMR-resistant K. pneumoniae strain was multidrug-resistant and may have possessed multiple resistance factors like penicillin-binding protein mutations and increased drug efflux pumps in addition to AmpC production; although these are possible factors for resistance in K. pneumoniae, it is important to note that imipenem is not subject to efflux, and therefore, efflux pumps would not be a mechanism of resistance for IMR. Overexpression or modification of drug efflux pumps and mutation of penicillin-binding protein have been reported as mechanisms of resistance to IMR (20); however, this mechanism is not definitive, as another study did not find evidence to support that imipenem or REL was subject to efflux (5). These mechanisms of resistance were not analyzed in the present study, and therefore, mechanisms of IMR resistance other than MBL in the studied isolates are unknown.

In the prospective study, antibacterial susceptibility was also measured for bacterial species listed in the IMI indication in Japan. The antibacterial activities of IMR were equal to or higher than those of imipenem against all bacterial species collected, and it was confirmed that the antibacterial activity of imipenem was not weakened by the combination with REL. There were imipenem-nonsusceptible isolates of P. mirabilis, P. vulgaris, and Providencia spp., but the enhancement of antibacterial activity of imipenem by REL was minimal. Since these species are known to be carbapenem-resistant by mechanisms other than β-lactamases (3, 21), REL was not effective in restoring imipenem susceptibility in these species. In *Morganellaceae*, enhancement of the antibacterial activity of imipenem by REL was observed only in M. morganii. With the exception of 1 strain, IMR-susceptible M. morganii strains did not show phenotypes of ESBL expression or constitutive expression of AmpC (data not shown). Since AmpC production is known to be one of the mechanisms of carbapenem resistance in M. morganii (22), REL may have restored the activity of imipenem by inhibiting AmpC induced by imipenem. Although IMI is indicated for infections due to Staphylococcus spp. and Enterococcus spp., the MIC_90_ values of IMR and imipenem against MRSA, MRSE, methicillin-resistant coagulase-negative Staphylococcus (MRCNS; other than S. epidermidis), E. faecium, and E. avium isolates were high. It is known that there are some strains of these species that are less susceptible to imipenem (23, 24), and the results of this study were consistent with those reports.

Comparison of the results of prospective studies with overseas surveillance revealed differences in the IMR susceptibilities of P. aeruginosa and Acinetobacter species isolates (10, 11). In the United States and Europe, about 10% of imipenem-nonsusceptible P. aeruginosa strains were resistant to IMR, while about 30% were resistant in this study. This difference is considered to be due to the higher proportion of MBL-possessing isolates in this study than in the United States (10) or Europe (11). The percentages of susceptibility of Acinetobacter spp. isolates to IMR were approximately 50% and 10% in the United States and Europe, respectively, while that observed in this study was 100%. The main mechanism of carbapenem resistance in Acinetobacter spp. is OXA-type carbapenemases, which are not inhibited by REL, and distribution of them is common overseas yet rare in Japan (25). This may account for the difference in the susceptibilities of Acinetobacter species isolates to IMR.

Several limitations should be considered when interpreting the results of this study. Primarily, there was a small number of imipenem-nonsusceptible isolates and the number of bacterial species was limited. Among the species listed in the indication of IMI/REL, imipenem-nonsusceptible isolates of Citrobacter spp. and Acinetobacter spp. were not collected in this study. However, a lower MIC_50/90_ of IMR than of imipenem was observed for Citrobacter spp., suggesting that IMR may be effective against imipenem-nonsusceptible Citrobacter spp. In addition, this study lacked the genetic analysis needed to confirm the presence of β-lactamases in phenotypically β-lactamase-positive isolates. Furthermore, resistance mechanisms other than β-lactamases were not analyzed.

In summary, IMR generally showed antibacterial activity against imipenem-nonsusceptible Enterobacterales other than *Morganellaceae* and P. aeruginosa isolates tested in this study, which often correspond to the indicated pathogens of IMI/REL in Japan. The antibacterial activities of IMR against imipenem-nonsusceptible isolates were lower than those of AMK and comparable to or greater than those of other drugs. The activity of IMR was superior to those of the reference drugs other than AMK and TGC for isolates with low susceptibility to imipenem, including isolates with AmpC or ESBL, which are 2 resistance mechanisms that are affected by REL. Although the efficacy was comparable to or less than those of existing drugs like AMK and TGC, issues with these drugs related to safety (26–28) and resistance (29–31) suggest that IMR is expected to be a new option for treatment of infections caused by carbapenem-resistant pathogens.

## MATERIALS AND METHODS

### Bacterial isolates.

In the prospective study, aerobic bacteria (*N* = 1,920 isolates, including Staphylococcus spp., Enterococcus spp., Streptococcus spp., Citrobacter spp., E. cloacae complex, E. coli, K. pneumoniae, Klebsiella oxytoca, K. aerogenes, S. marcescens, M. morganii, P. mirabilis, P. vulgaris, Providencia spp., P. aeruginosa, Acinetobacter spp., Burkholderia cepacia complex, and H. influenzae) and anaerobic bacteria (*N* = 75 isolates, including Peptostreptococcus spp., Bacteroides spp., and Prevotella spp.) were isolated from various clinical specimens of patients in 605 medical institutions throughout Japan from January 2016 to September 2016 by LSI Medience Corporation. Identification of the isolates was carried out in accordance with the *Manual of Clinical Microbiology* (32), and strains were frozen at −70°C in skim milk. Collection of these strains was conducted in compliance with the “Ethical Guidelines for Epidemiologic Research” issued by the Ministry of Education, Culture, Sports, Science and Technology and Ministry of Health, Labor and Welfare (33) and the notification of the Japanese Society for Clinical Microbiology.

In the antibacterial susceptibility test of bacterial stocks, imipenem-nonsusceptible strains of E. coli, E. cloacae complex, and K. pneumoniae (*N* = 8) were collected by Toho University from 6 hospitals in Japan between 2013 and 2016 and frozen at −80°C in 15% glycerol. Identification of bacterial species was performed using draft whole-genome sequencing as described previously (34).

### Antibacterial susceptibility testing.

Following the CLSI guidelines (3, 35, 36), the broth microdilution method and agar dilution method were used for aerobic bacteria and anaerobic bacteria, respectively. Ninety-six-well frozen plates (Eiken Chemical Co., Ltd., Tokyo, Japan) containing drugs were used for testing. All isolates were tested against IMR (REL was fixed at 4 μg/mL), imipenem, MEM, TZP (tazobactam was fixed at 4 μg/mL), CAZ, FEP, LVX, AMK, CST, and TGC. The concentration range of drugs other than CST was 0.06 to 128 μg/mL, and that of CST was 0.06 to 32 μg/mL. MICs were interpreted as susceptible, intermediate, and resistant using CLSI breakpoints (3). MIC values for IMR and TZP are presented for imipenem and piperacillin, respectively.

### Detection of β-lactamases.

In the prospective study, E. coli, Klebsiella species, and P. mirabilis isolates were tested for ESBLs, and Enterobacterales, P. aeruginosa, and Acinetobacter species isolates were tested for MBL and AmpC. An MIC of ≥2 μg/mL for CAZ or cefotaxime (CTX) and an ≥8-fold decrease in the MIC of CAZ or CTX in combination with clavulanic acid versus that of CAZ or CTX alone was considered positive for ESBLs. An MIC of ≥2 μg/mL for CAZ or CTX, an ≥8-fold decrease in the MIC of CAZ or CTX in combination with 3-aminophenylboronic acid versus that of CAZ or CTX alone, and a negative result in the modified carbapenem inactivation method (3) was considered positive for constitutive expression of AmpC (37). Unlike imipenem (38), both CAZ and CTX are not strong inducers of AmpC (37); therefore, this test only detected constitutive production of AmpC. Since many species of bacteria encode a chromosomal AmpC (e.g., E. cloacae and P. aeruginosa) and since imipenem is a strong inducer of AmpC, all members of these species should be considered to hyperproduce AmpC when imipenem is present, either in an *in vitro* susceptibility test or in a patient. A decrease of ≥8-fold in the MIC of CAZ or imipenem in combination with dipicolinic acid versus that of CAZ or imipenem alone was considered positive for MBL. The final concentrations of clavulanic acid, 3-aminophenylboronic acid, and dipicolinic acid were fixed at 4 μg/mL, 200 μg/mL, and 175 μg/mL, respectively.

In the antibacterial susceptibility test using bacterial stocks, the β-lactamase gene profile was identified using draft whole-genome sequencing as described in a previous study (34). Briefly, bacterial DNA libraries were sequenced on a MiSeq system. Acquired antibacterial resistance genes were identified using the ResFinder database, version 2.1.

### Determination of antibacterial-resistant strains.

S. aureus was defined as methicillin-susceptible S. aureus when the MIC of OXA was ≤2 μg/mL and as MRSA when the MIC of OXA was ≥4 μg/mL. S. epidermidis was defined as methicillin-susceptible S. epidermidis when the MIC of OXA was ≤0.25 μg/mL and as MRSE when the MIC of OXA was ≥0.5 μg/mL. Coagulase-negative Staphylococcus was defined as methicillin-susceptible coagulase-negative Staphylococcus (MSCNS) when the MIC of OXA was ≤0.25 μg/mL and as methicillin-resistant coagulase-negative Staphylococcus (MRCNS) when the MIC of OXA was ≥0.5 μg/mL. Streptococcus pneumoniae was defined as penicillin-susceptible S. pneumoniae when the MIC of penicillin G was ≤0.06 μg/mL and as penicillin-resistant S. pneumoniae when the MIC of penicillin G was ≥2 μg/mL. β-Lactamase–negative H. influenzae with an ampicillin MIC of ≥2 μg/mL was classified as β-lactamase–negative ampicillin-resistant H. influenzae (BLNAR). β-Lactamase activity was detected by the nitrocefin spot plate method.

### Data availability.

The data sharing policy of Merck Sharp & Dohme Corp., a subsidiary of Merck & Co., Inc., Kenilworth, NJ, USA, including restrictions, is available at http://engagezone.msd.com/ds_documentation.php. Requests for access to the clinical study data can be submitted through the EngageZone site or via email to dataaccess@merck.com.
